# miR-431 inhibits adipogenic differentiation of human bone marrow-derived mesenchymal stem cells via targeting insulin receptor substance 2

**DOI:** 10.1186/s13287-018-0980-4

**Published:** 2018-08-30

**Authors:** Yangling Wang, Lei Yang, Xiaofeng Liu, Tao Hong, Tao Wang, Aiwu Dong, Jiangxiong Li, Xiaoyuan Xu, Lingling Cao

**Affiliations:** 1 Department of Endocrinology, The First Hospital of Jiujiang City, Jiujiang, 332000 China; 2 Jiujiang Affiliated Hospital of Nanchang University, Jiujiang, 332000 China; 3 Key Laboratory of System Bio-medicine of Jiangxi Province, Jiujiang University, Jiujiang, 332000 China

**Keywords:** Adipogenic differentiation, Bone marrow-derived mesenchymal stem cells, miR-431, IRS2

## Abstract

**Background:**

An understanding of the mechanism underlying adipogenic differentiation of human bone marrow-derived mesenchymal stem cells (hMSCs) will provide new therapeutic approaches for many diseases, including osteoporosis. This study aimed to investigate the role of miR-431 in adipogenic differentiation of hMSCs.

**Methods:**

hMSCs were induced for adipogenic differentiation and miR-431 was detected by polymerase chain reaction (PCR). hMSCs were transfected by miR-431 or small interfering RNA (siRNA) for insulin receptor substance 2 (IRS2). The expression of IRS2 was detected by PCR and Western blot analysis. The targeting of the 3′-untranslated region (UTR) of IRS2 by miR-431 was examined by luciferase assay.

**Results:**

miR-431 expression was decreased during adipogenesis of hMSCs. Overexpression of miR-431 inhibited adipogenic differentiation, accompanied by the downregulation of CCAAT/enhancer binding protein α (C/EBPα) and peroxisome proliferator-activated receptor γ (PPARγ), two key regulators of adipogenesis. Moreover, miR-431 decreased both protein and mRNA levels of IRS2. The expression of IRS2 was increased during adipogenic differentiation of hMSCs in conjunction with decreased levels of miR-431, and knockdown of IRS2 in hMSCs inhibited adipogenic differentiation. Luciferase assay confirmed that miR-431 targeted the 3′-UTR of IRS2 in hMSCs.

**Conclusions:**

This is the first study to show that miR-431 inhibits adipogenic differentiation of hMSCs via targeting IRS2.

## Background

Human bone marrow-derived mesenchymal stem cells (hMSCs) are multipotent stem cells in the bone marrow with the capacity for self-renewal and multilineage differentiation, including osteogenesis, chondrogenesis, and adipogenesis [1]. The balance between osteogenic and adipogenic differentiation of hMSCs is disrupted under in many human diseases such as osteoporosis, age-related bone loss, and osteonecrosis [2, 3]. In addition, abnormal fat accumulation increases the risk of life-threatening diseases such as type 2 diabetes, atherosclerosis, and cancer [4, 5]. Therefore, understanding the molecular mechanisms of adipogenic differentiation of hMSCs may provide new strategies for the prevention and treatment of these diseases.

Adipogenesis is a well-orchestrated multistep process in which a sequential activation of transcription factors leads to the formation of mature adipocytes, especially CCAAT/enhancer binding protein α (C/EBPα) and peroxisome proliferator-activated receptor γ (PPARγ) [6]. PPARγ forms heterodimers with the retinoid X receptor (RXR) and activates lipogenic genes such as adipocyte fatty acid-binding protein (aP2), FAS, and glucose transporter 4 (GLUT4) to initiate adipogenesis in exponentially growing fibroblast cell lines. C/EBPα activates a variety of adipocyte-specific genes and initiates the differentiation of preadipocytes by inducing PPARγ expression during adipogenic differentiation. In addition, insulin receptor substance 2 (IRS2), which acts as a major common substrate for insulin and insulin-like growth factor 1 (IGF-1) receptor tyrosine kinases, plays an important role in adipogenic differentiation [7].

MicroRNAs (miRNAs) are a group of small noncoding RNAs that mainly function as post-transcriptional regulators of gene expression and control cell proliferation, apoptosis, and tumorigenesis [8, 9]. Additionally, miRNAs have been shown to participate in the regulation of adipogenic and osteogenic differentiation of hMSCs [10–14]. A previous study reported that miR-143 promoted adipogenic differentiation via the targeting of extracellular regulated protein kinases 5 (ERK5) in human preadipocytes [15]. Our preliminary microarrays showed that miR-431 was significantly downregulated and IRS2 was upregulated during adipogenic differentiation of hMSCs, consistent with the prediction of IRS2 as a target of miR-431 [16].

Therefore, in this study we focused on miR-431 and investigated its role during adipogenesis of hMSCs. We found that the expression of miR-431 was decreased during adipogenic differentiation of hMSCs in conjunction with increased levels of IRS2. Overexpression of miR-431 suppressed the expression of PPARγ, C/EBPα, and IRS2, and inhibited adipogenic differentiation. Target prediction analyses revealed IRS2 as a putative miR-431 target with a conserved miR-431 binding site, and luciferase reporter assay demonstrated a functional miR-431 response element in the 3′-untranslated region (UTR) of IRS2. These results suggest that miR-431 functions as a repressor of human adipogenesis by directly targeting IRS2.

## Methods

### Cell culture

hMSCs were purchased from Cyagen Biosciences (Guangzhou, China) and identified as described previously [17]. The cells were cultured in α-minimum essential medium (α-MEM; Gibco) supplemented with 10% fetal bovine serum (FBS; Gibco), 100 IU/ml penicillin, and 100 μg/ml streptomycin in a humidified atmosphere of 5% CO_2_ at 37 °C.

### Adipogenic differentiation

Adipogenic differentiation was performed as described previously [17]. Cells were seeded into six-well plates at a density of 1 × 10^4^ cells/ml. Upon reaching confluency, hMSCs were stimulated in adipogenic medium composed of α-MEM supplemented with 10% FBS, 1 μM dexamethasone, 0.5 mM isobutylmethylxanthine, and 0.01 mg/ml insulin for 7, 14, and 21 days. Adipogenesis was determined by Oil Red O staining and the induced expression of PPARγ and C/EBPα.

### Oil Red O staining

Oil Red O staining was conducted as described previously [13]. Cells were washed with phosphate-buffered saline (PBS) three times and fixed with 10% formalin for 5 min at room temperature. After fixation, cells were stained with filtered Oil Red O solution (stock solution: 0.5 g Oil Red O, Sigma-Aldrich, in 100 ml isopropanol; working solution: 60% Oil Red O stock solution and 40% distilled water) for 15 min at room temperature.

### Lentivirus infection and screening

miR-431 lentiviral vectors and IRS2-small interfering RNA (siRNA) lentiviral vector and negative control lentiviral vectors were purchased from Genechem (Shanghai, China). hMSCs were seeded into six-well plates, cultured to 20–30% confluence, and infected by 1 × 10^8^ TU/ml lentivirus diluted in 5 μg/ml polybrene and complete medium. After 10 h, the medium was refreshed and the cells were cultured for an additional 72 h. The medium containing 0.5 μg/ml puromycin was used to screen for cells with successful transduction of lentivirus.

### Luciferase reporter assay

Human IRS2 3′-UTR fragments containing putative binding sites for miR-431 were constructed by Genechem (Shanghai, China). Briefly, IRS2 3′-UTR was amplified by polymerase chain reaction (PCR) from human genomic DNA. Positions 3–6 of the seed match were mutated by overlap extension PCR. The fragments were cloned into a pmiRGLO reporter vector (Promega) downstream of the luciferase gene to generate the recombinant vectors pmiRGLO-IRS2-WT and pmiRGLO-IRS2-MUT. We plated 2 × 10^4^ HEK293 cells (purchased from American Type Culture Collection, Manassas, VA, USA) in a 96-well plate and, 20 h later, transfections were performed using 0.2 μl DharmaFECT Duo (Dharmacon), 80 ng p2-IRS2 or p2-IRS2-mut, and either 50 nM nontargeting control (NC) or miR-431. Cells were harvested 48 h later for the measurement of luciferase activity using a kit (Promega) following the manufacturer’s instructions.

### Quantitative real-time PCR (qRT-PCR) analysis

Total RNA was extracted from cells using Trizol reagent (Life Technologies) following the manufacturer’s instructions. First-strand cDNA was synthesized using a PrimeScript® RT reagent Kit (Life Technologies) according to the manufacturer’s instructions. qRT-PCR was performed using an ABI StepOnePlus Real-time Detection System (AB, CA, USA) and SYBR Green qPCR SuperMix (Invitrogen, USA). U6 small nuclear RNA and β-actin were used as internal controls. The primers used for reverse transcription and qRT-PCR are listed in Table 1. Each experiment was repeated independently at least three times, and the fold change in the expression of each gene was analyzed using the 2^–ΔΔ^CT method.

**Table 1:** Primer sequences used for quantitative real-time polymerase chain reaction

Gene	Accession No.	Forward (5′–3′)	Reverse (5′–3′)
*IRS2*	NM_003749	CCACAGTTCCGAGACCTTCT	TCGCTGCTTTTCCTGAGAGA
*C/EBPα*	NM_004364	CCAAGAAGTCGGTGGACAAGAAC	CACCTTCTGCTGCGTCTCCA
*PPARγ*	NM_015869	GGGATGTCTCATAATGCCATCAG	GCCCTCGCCTTTGCTTTG
*GAPDH*	NM_002046	ACCCACTCCTCCACCTTTG	CTCTTGTGCTCTTGCTGGG

### Western blot analysis

Cells were lysed in lysis buffer (150 mM NaCl, 1% NP40, and 50 mM Tris–HCl, pH 8.0) supplemented with protease inhibitors (2 μg/ml leupeptin, 2 μg/ml pepstain, 2 μg/ml aprotinin, and 2 μg/ml PMSF) on ice for 30 min; the lysate was centrifuged at 12,000 rpm for 20 min and the supernatant was stored at −20 °C. The sample was separated by 12% SDS-PAGE and electrotransferred onto PVDF membranes. After incubation in blocking buffer for 1 h at 37 °C, the membranes were incubated overnight at 4 °C with antibodies for IRS2 (Abcam; 1:500 dilution), C/EBPα (Abcam; 1:1000 dilution), PPARγ (Abcam; 1:1000 dilution), and β-actin (Abcam; 1:1000 dilution). After washing, the membranes were incubated with secondary antibody conjugated to horseradish peroxidase at 37 °C for 30 min. Finally, immunoreactive bands were visualized using a Super Signal West Pico kit (Pierce) according to the manufacturer’s instructions.

### Statistical analysis

All experiments were repeated at least three times and the data are presented as the mean ± SEM. The data were analyzed by analysis of variance (ANOVA), followed by Fisher’s least significant difference test and independent samples Student’s *t* test, using SPSS software version 13.0 (SPSS, Chicago, IL, USA).

## Results

### miR-431 is downregulated during adipogenesis of hMSCs

**Fig. 1:**
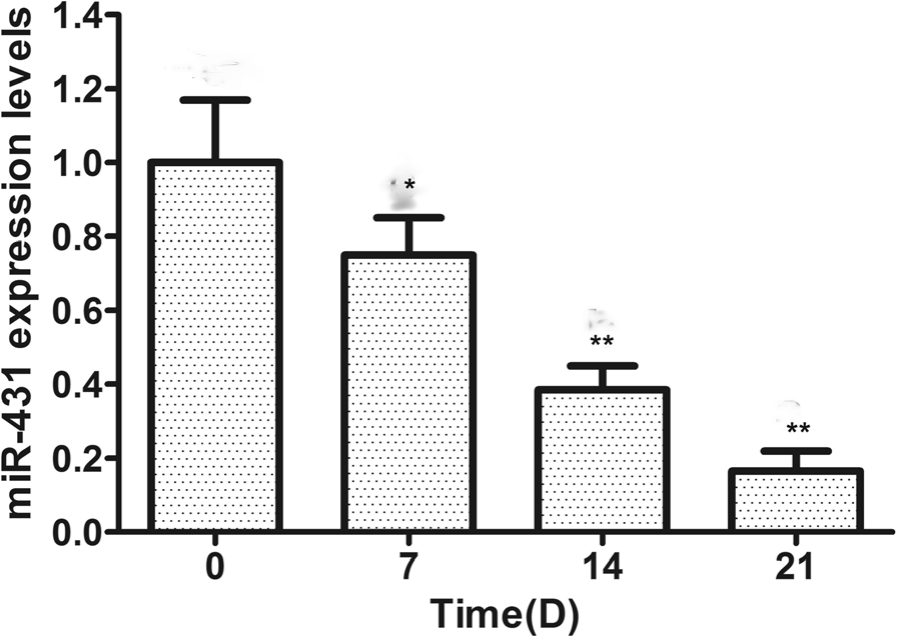
The expression levels of miR-431 during adipogenesis of hMSCs. Data are presented as mean ± SEM (*n* = 3). *p < 0.05, **p < 0.01, versus day 0

To investigate the role of miR-431 during adipogenesis of hMSCs, we first examined the expression of miR-431 during adipogenesis of hMSCs at different stages (0, 7, 14, and 21 day). We found that the expression of miR-431 decreased by 30% after 7 days of culture and remained at low levels until 21 days (Fig. 1).

### miR-431 inhibits adipogenic differentiation of hMSCs

**Fig. 2:**
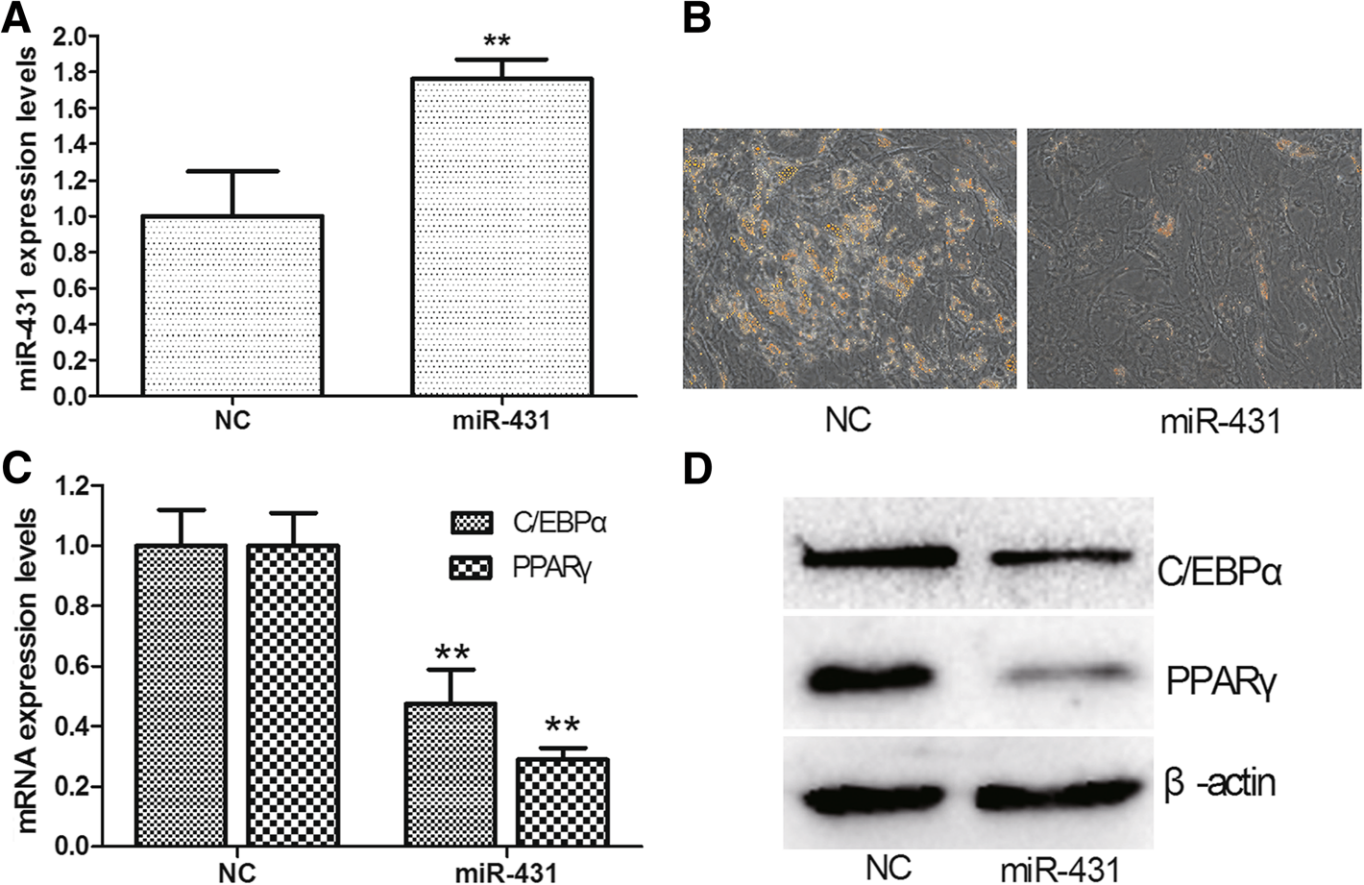
miR-431 inhibits adipogenesis of hMSCs. **a** The expression levels of miR-431 in hMSCs infected by miR-431 lentivirus or negative control (NC) lentivirus. **b** Oil Red O staining of hMSCs infected by miR-431 lentivirus or NC lentivirus. **c** The mRNA expression levels of CCAAT/enhancer binding protein α (C/EBPα) and peroxisome proliferator-activated receptor γ (PPARγ) in hMSCs infected by miR-431 lentivirus or NC lentivirus. **d** The protein expression levels of C/EBPα and PPARγ in hMSCs infected by miR-431 lentivirus or NC lentivirus. Data are presented as the mean ± SEM (*n* = 3). **p < 0.01, versus NC

To determine the role of miR-431 during adipogenesis, we used lentivirus to overexpress miR-431 in hMSCs (Fig. 2a). Oil Red O staining showed that miR-431 overexpression led to decreased adipogenic differentiation (Fig. 2b). In addition, Western blot and qRT-PCR analysis showed that the expression levels of the adipogenic markers C/EBPα and PPARγ were significantly lower in hMSCs overexpressing miR-431 (Fig. 2c, d).

### IRS2 is a target of miR-431

**Fig. 3:**
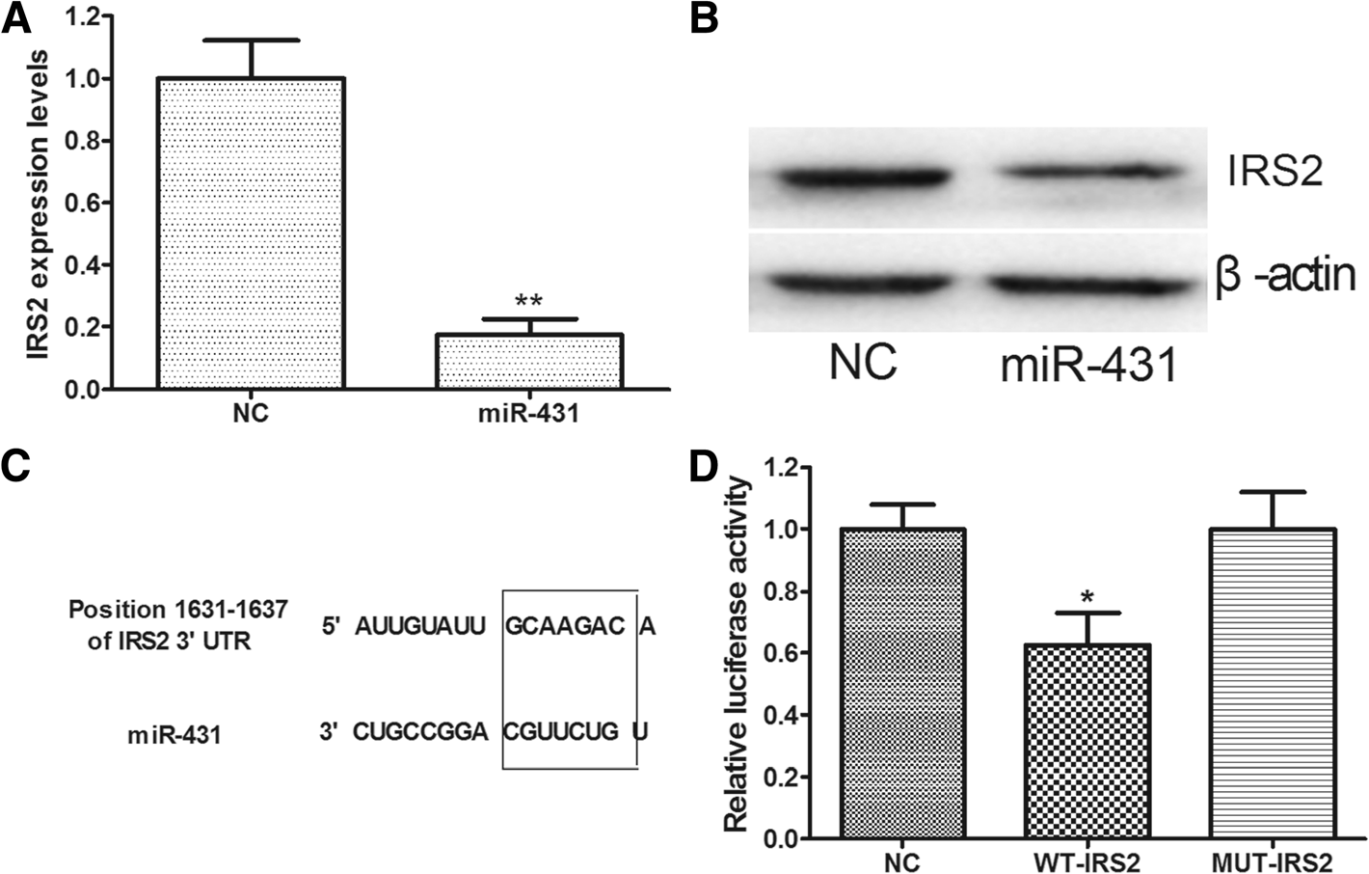
miR-431 targets the 3′-UTR of IRS2. **a** The mRNA expression levels of insulin receptor substance 2 (IRS2) in hMSCs infected by miR-431 lentivirus or negative control (NC) lentivirus. **b** The protein expression levels of IRS2 in hMSCs infected by miR-431 lentivirus or NC lentivirus. **c** The matching of miR-431 with IRS2 3’-untranslated regions (UTRs). **d** Luciferase activity assay; HEK293 cells were infected by miR-431 lentivirus or NC lentivirus and transfected with wild-type (WT) or mutant (MUT) IRS2 3′-UTR reporter. Data are presented as the mean ± SEM (*n* = 3). *p < 0.05, **p < 0.01, versus NC

To understand the mechanism by which miR-431 inhibits adipogenic differentiation of hMSCs, we aimed to characterize the targets of miR-431. IRS2 was predicted to be a target of miR-431 [16]. As expected, mRNA and protein expression levels of IRS2 were decreased in hMSCs infected by miR-431 lentivirus compared with cells infected by NC lentivirus (Fig. 3a, b). Based on TargetScan, a miR-431 targeting site was predicted in the 3′-UTR of IRS2 (Fig. 3c). Luciferase reporter assay showed that luciferase activity mediated by 3′-UTR of IRS2 decreased significantly in HEK293 cells infected by miR-431 lentivirus compared with cells infected by NC lentivirus, but site-directed mutagenesis of the seed region in 3′-UTR of IRS2 abolished the inhibitory effect by miR-431 lentivirus (Fig. 3d). Collectively, these data indicate that miR-431 inhibits IRS2 expression by targeting its 3′-UTR.

### IRS2 is upregulated during adipogenic differentiation of hMSCs

**Fig. 4:**
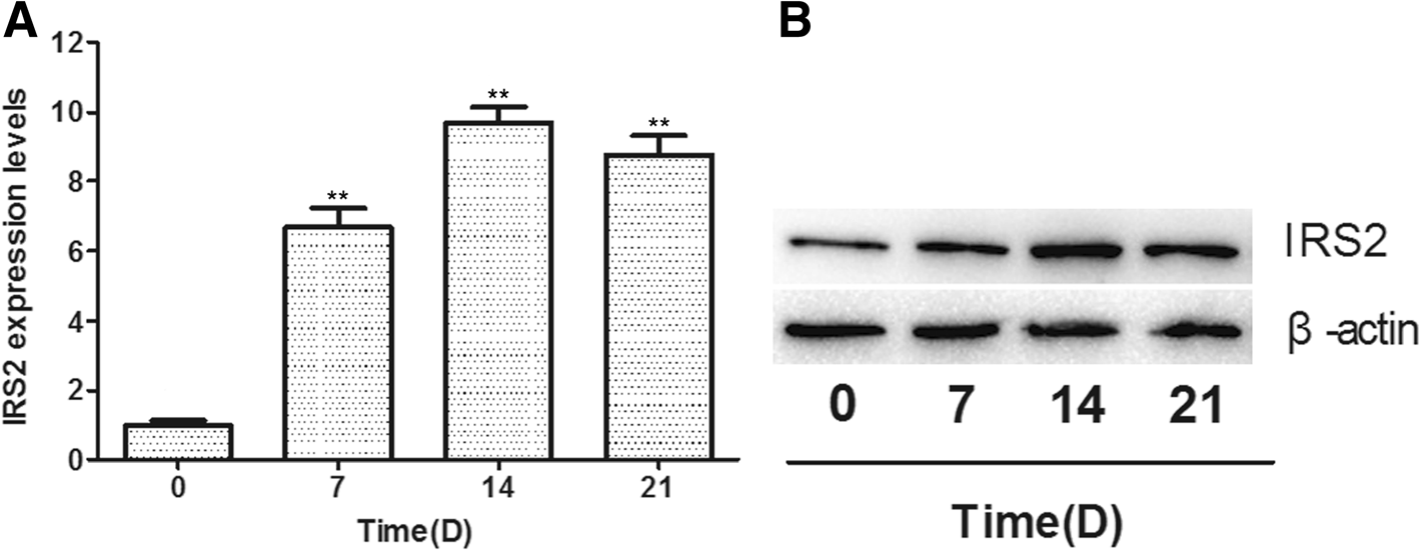
The expression levels of IRS2 during adipogenesis of hMSCs. **a** The mRNA expression levels of insulin receptor substance 2 (IRS2) during adipogenesis of hMSCs. **b** The protein expression levels of IRS2 during adipogenesis of hMSCs. Data are presented as the mean ± SEM (*n* = 3). **p < 0.01, versus day 0

Next, we examined the role of IRS2 in the regulation of adipogenic differentiation. We analyzed the expression of IRS2 by qRT-PCR and Western blot analysis. The results showed that mRNA and protein levels of IRS2 were increased during adipogenesis of hMSCs (Fig. 4a, b).

### IRS2 knockdown inhibits adipogenic differentiation of hMSCs

**Fig. 5:**
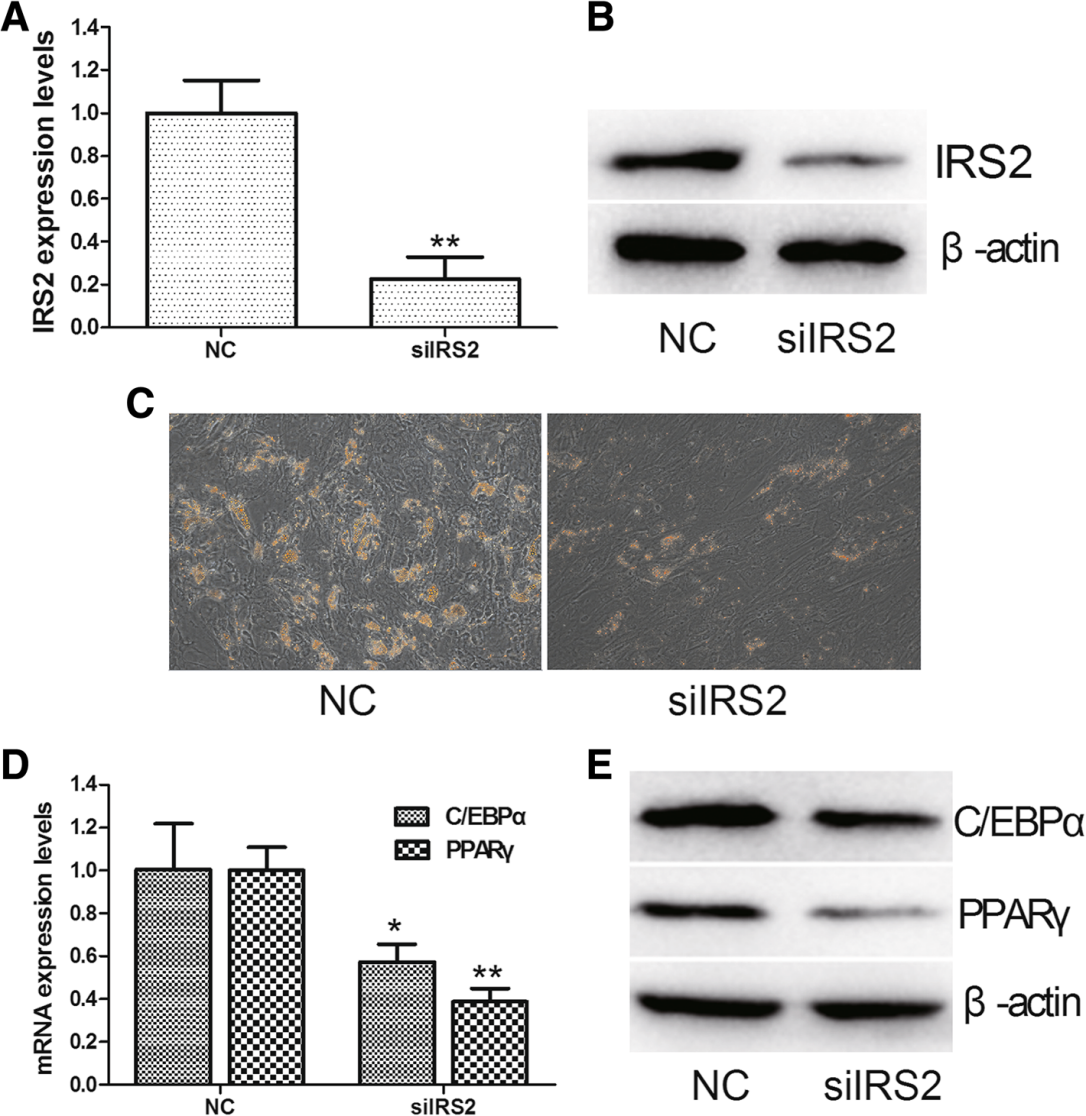
IRS2 promotes adipogenesis of hMSCs. **a** The mRNA expression levels of insulin receptor substance 2 (IRS2) in hMSCs infected by IRS2 small interfering RNA (siRNA) lentivirus or negative control (NC) lentivirus. **b** The protein expression levels of IRS2 in hMSCs infected by IRS2 siRNA lentivirus or NC lentivirus. **c** Oil Red O staining of hMSCs infected by IRS2 siRNA lentivirus or NC lentivirus. **d** The mRNA expression levels of CCAAT/enhancer binding protein α (C/EBPα) and peroxisome proliferator-activated receptor γ (PPARγ) in hMSCs infected by IRS2 siRNA lentivirus or NC lentivirus. **e** The protein expression levels of C/EBPα and PPARγ in hMSCs infected by IRS2 siRNA lentivirus or NC lentivirus. Data are presented as the mean ± SEM (*n* = 3). *p < 0.05, **p < 0.01, versus NC

Since IRS2 is upregulated during adipogenic differentiation of hMSCs, we employed siRNA lentivirus to knockdown IRS2 in hMSCs. qRT-PCR and Western blot analysis confirmed that mRNA and protein levels of IRS2 were significantly decreased in hMSCs infected by IRS2 siRNA lentivirus compared with NC lentivirus (Fig. 5a, b). Furthermore, knockdown of IRS2 significantly suppressed adipogenic differentiation on day 14, as indicated by Oil Red O staining (Fig. 5c). In addition, knockdown of IRS2 led to the downregulation of the adipogenic markers C/EBPα and PPARγ at both the mRNA and protein level (Fig. 5d, e).

### IRS2 rescues adipogenic differentiation of hMSCs inhibited by miR-431

**Fig. 6:**
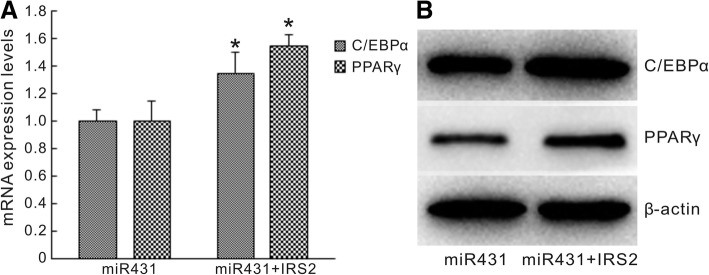
IRS2 rescues the adipogenesis of hMSCs inhibited by miR-431. **a** The mRNA expression levels of CCAAT/enhancer binding protein α (C/EBPα) and peroxisome proliferator-activated receptor γ (PPARγ) in hMSCs infected by miR-431 lentivirus alone (miR431) or combined with insulin receptor substance 2 (IRS2) expression vector (miR431 + IRS2). **b** The protein expression levels of C/EBPα and PPARγ in hMSCs infected by miR-431 lentivirus alone (miR431) or combined with IRS2 expression vector (miR431 + IRS2). Data are presented as the mean ± SEM (*n* = 3). *p < 0.05, versus miR-431 lentivirus alone

To confirm the functional interaction between IRS2 and miR-431 during adipogenic differentiation of hMSCs, we overexpressed IRS2 in hMSCs. qRT-PCR and Western blot analysis showed that overexpression of IRS2 led to the upregulation of the adipogenic markers C/EBPα and PPARγ at both the mRNA and protein level (Fig. 6a, b).

## Discussion

An increasing number of studies have shown the involvement of miRNAs in the regulation of adipogenic differentiation of mesenchymal stem cells, such as miR-143, miR-204, miR-320, miR-637, miR-378, and miR-155 [18–21]. However, the role of miR-431 in adipogenic differentiation of mesenchymal stem cells has not been investigated. In the present study, we provide the first evidence that miR-431 functions as a negative regulator of adipogenesis of hMSCs by targeting IRS2.

We first detected the pattern of miR-431 expression during adipogenesis of hMSCs at different stages, and found that the miR-431 level was significantly decreased during adipogenesis of hMSCs. Overexpression of miR-431 in hMSCs impaired adipogenic differentiation as indicated by Oil Red O staining and decreased expression levels of C/EBPα and PPARγ, which are adipogenic differentiation markers [22]. These results indicate that miR-431 negatively regulates adipogenesis of hMSCs.

Previous studies have indicated that the IGF-1 signaling pathway plays an important role in adipogenic differentiation [23]. It has been reported that IRS1 and IRS2, cytoplasmic signaling molecules that mediates the effects of the IGF-1 signaling pathway, play a crucial role in adipogenic differentiation [7]. Accordingly, we analyzed the expression of IRS2 in adipogenic differentiation by qRT-PCR and Western blot analysis and found that the expression of IRS2 increased during adipogenic differentiation of hMSCs. In addition, knockdown of IRS2 impaired the adipogenic differentiation of hMSCs and inhibited the expression of the adipogenic markers C/EBPα and PPARγ. Our findings further support previous reports that the IGF-1 signaling pathway is important in adipogenesis [7, 24].

Since IRS2 expression is negatively correlated with the miR-431 level during adipogenic differentiation of hMSCs, and since a miR-431 complementary site has been predicted in the 3′-UTR of IRS2 [16], we speculated that IRS2 is a target of miR-431. Indeed, we found that overexpression of miR-431 led to decreased expression of IRS2 at the mRNA and protein level. Furthermore, we performed luciferase reporter assay with luciferase reporter vectors containing wild-type or mutant IRS2 3′-UTR. The results indicated that IRS2 is a direct target of miR-431. In addition, we found that IRS2 was upregulated during adipogenic differentiation of hMSCs, and knockdown of IRS2 inhibited adipogenic differentiation of hMSCs. Furthermore, the overexpression of IRS2 rescued adipogenic differentiation of hMSCs inhibited by miR-431.

## Conclusion

This is the first study to report that miR-431 negatively regulates adipogenic differentiation of hMSCs. In addition, we demonstrated that IRS2 is a target of miR-431 during adipogenesis of hMSCs, and IRS2 is upregulated and promotes adipogenic differentiation of hMSCs. These findings suggest that miR-431 inhibits adipogenic differentiation of hMSCs by targeting IRS2. Therefore, miR-431 is a promising target to modulate the balance between osteogenic and adipogenic differentiation of hMSCs and provides novel therapeutic approaches for osteoporosis and diabetes.

## Abbreviations

C/EBPα: CCAAT/enhancer binding protein α
hMSC: Human bone marrow-derived mesenchymal stem cell
IGF-1: Insulin-like growth factor 1
IRS2: Insulin receptor substance 2
miRNA: MicroRNA
NC: Nontargeting control
PCR: Polymerase chain reaction
PPARγ: Peroxisome proliferator-activated receptor γ
qRT-PCR: Quantitative real-time polymerase chain reaction
siRNA: Small interfering RNA
UTR: Untranslated region
