# Correction to: miR-431 inhibits adipogenic differentiation of human bone marrow-derived mesenchymal stem cells via targeting insulin receptor substance 2

**DOI:** 10.1186/s13287-019-1173-5

**Published:** 2019-02-21

**Authors:** Yanling Wang, Lei Yang, Xiaofeng Liu, Tao Hong, Tao Wang, Aiwu Dong, Jiangxiong Li, Xiaoyuan Xu, Lingling Cao

**Affiliations:** 1 Department of Endocrinology, The First Hospital of Jiujiang City, Jiujiang, 332000 China; 2 Jiujiang Affiliated Hospital of Nanchang University, Jiujiang, 332000 China; 3 Key Laboratory of System Bio-medicine of Jiangxi Province, Jiujiang University, Jiujiang, 332000 China

**Correction to: Stem Cell Res Ther (2018) 9:231**

**https://doi.org/10.1186/s13287-018-0980-4**

The original article [1] contains an error in spelling of author, Yanling Wang’s name. The correct version can instead be viewed in this Correction article.
